# Reliability assessment of agricultural sensors evaluated through algal coverage in hydroponic tomato production systems

**DOI:** 10.1038/s41598-026-38555-y

**Published:** 2026-02-12

**Authors:** Saksonita Khoeurn, No Hyeon Park, Hye Kyoung Jahng, Jaehyuk Jeon, Seungback Jung, Wan-Sup Cho, Yumin Joung, Eunji Lee, Dong Sub Kim

**Affiliations:** 1 BigDataLabs Co., Ltd., Cheongju, 28644 South Korea; 2 Department of Big Data, Chungbuk National University, Cheongju, 28644 South Korea; 3 Department of Management Information Systems, Cheongju, 28644 South Korea; 4 eney Co., Ltd., Seoul, 08513 South Korea; 5 WHYBIZ Corp, Seoul, 06978 South Korea; 6 Department of Horticulture, Kongju National University, Yesan, 32439 South Korea

**Keywords:** Hydroponic, Rockwool, *Solanum lycopersicum*, Sensor reliability, Plant sciences, Ecology, Environmental sciences, Engineering

## Abstract

In modern agricultural systems, hydroponics represents a crucial advancement in integrating digital technologies and precision farming practices for sensor-mediated cultivation. These systems employ continuous environmental monitoring to enhance operational efficiency and promote plant growth. However, environmental factors and technical issues can undermine data integrity because of faulty sensor performance. Detecting sensor malfunctions during cultivation is challenging. This study investigated whether algal coverage patterns on sensor surfaces could explain observed variations in sensor-recorded environmental parameters in rockwool-based hydroponic tomato systems. In a controlled greenhouse setting, 117 environmental sensors continuously monitored root-zone temperature, relative humidity, pH, and electrical conductivity (EC). Despite uniform conditions, substantial sensor data variation was observed. Post-cultivation analysis revealed marked differences in algal coverage across sensor surfaces. We hypothesized that algae coverage ratios reflect differential nutrient solution distribution within rockwool substrate, potentially explaining sensor data variation. After 3 months, 39 sensors were categorized according to their algal coverage: 22 sensors exhibited high colonization (≥ 90% coverage) and 17 displayed minimal coverage (< 10%). The sensors with significant algal coverage presented markedly elevated substrate relative humidity (85.6% vs. 41.9%) and EC values (0.77 dS/m vs. 0.33 dS/m) than those with minimal coverage. The tomato productivity metrics did not differ significantly, implying potential biological adaptations to water deficiency or hydrodynamic characteristics of the rockwool. These findings suggest that quantifying algal growth may indirectly reflect environmental parameters, particularly relative humidity and EC, while serving as an inferential validation indicator of sensor data reliability in hydroponic systems. This study addresses the crucial challenges in achieving precise environmental monitoring in advanced agricultural systems, optimizing resource utilization, and improving crop production efficiency.

**Supplementary Information:**

The online version contains supplementary material available at 10.1038/s41598-026-38555-y.

## Introduction

Tomatoes (*Solanum lycopersicum*) rank among the most widely cultivated and consumed vegetables globally^1^. Inadequate water management can affect fruit quality in crops with high water requirements (e.g., tomatoes), which is why hydroponics are commonly used for tomato cultivation^2,3^. Hydroponics is a cultivation method that eliminates the need for soil and supplies water and nutrients directly to plant roots, thereby maximizing resource efficiency in agricultural practices. This method mitigates pest and disease risks, conserves water, and optimizes space utilization, making it widely used in modern agriculture^4,5^. In particular, the growth medium used in hydroponics is essential for stabilizing plant roots and ensuring adequate moisture and oxygen, thereby considerably influencing crop performance^6,7^.

Rockwool is a widely used artificial medium in hydroponics because of its light weight, ease of handling, and physical and chemical stability, making it an ideal choice^8^. The porous structure of rockwool enables simultaneous storage of both water and air, thereby facilitating an environment conducive to adequate oxygen supply for plant roots. Additionally, controlling the pH and electrical conductivity (EC) of the nutrient solution is relatively straightforward, enabling the effective management of environmental variables essential for plant growth^9^.

In Rockwool substrates, a nutrient solution is typically supplied via drip fertigation, wherein drippers dispense a fixed volume of solution that gradually spreads from the point of application throughout the rockwool^8,9^. The area around the drippers typically retains a relatively high moisture content and nutrient salts and fosters conditions conducive to algal growth^10^. Algal formation arises from abundant nutrients, particularly nitrogen and phosphorus, in the solution in conjunction with excessive light exposure^11^. Conversely, the dry areas of the rockwool, where the nutrient mixture is insufficient, present a lower risk of algal growth^12^. By observing the location and extent of algal formation, we can indirectly infer the flow of the nutrient solution within the substrate.

Modern agriculture is evolving into various forms, including digital, smart, and precision farming, with hydroponic systems representing a key application of these technologies. Data-driven, digitized agricultural models, contribute to increased efficiency and productivity by automating the hydroponic environment. Various sensors are employed to monitor environmental variables, including pH, EC, temperature, and humidity, in real-time, thereby facilitating the maintenance of optimal conditions for plant growth.

Nevertheless, sensors may experience errors owing to system malfunctions or environmental factors, potentially compromising data reliability^13,14^. For example, despite issues with sensor data, crop yields may remain unaffected. This phenomenon is often attributed not to errors in the sensors themselves but to the specific characteristics of the growing environment that compensate for such issues. Therefore, precise sensor data management and comprehensive analysis of environmental variables are essential to enhance the reliability of hydroponic systems.

In this study, we aimed to investigate the effect of algal formation on sensor data reliability in rockwool hydroponic tomato cultivation. Specifically, we analyzed the correlation between algal coverage and sensor data accuracy and explored strategies for enhancing data reliability and system efficiency. This study addresses the critical challenge of ensuring accurate and reliable data collection in hydroponic systems, which is vital for optimizing resource management and crop production.

## Materials and methods

The study framework employed to assess sensor reliability in hydroponic tomato cultivation is depicted in Fig. 1. The diagram illustrates the complete workflow from sensor installation to data analysis. Each of the 117 tomato plants was equipped with a sensor to monitor Moisture, pH, EC, and temperature (HWASUBUN-Fork, WHYBIZ Corp, Seoul, South Korea) in the root zone. Three sensors were connected to each wireless node that transmitted data via a gateway to a centralized database at 10-min intervals over the 3-month cultivation period (July 18–October 16, 2024). The sensors were systematically arranged and numbered (1–117) between the front and back gates of a greenhouse. The algal coverage on each sensor was photographically documented using a smartphone (Galaxy A32, Samsung, Seoul, South Korea) after the last harvest was finished and quantified using quadrant analysis on images processed to present only the rockwool block surface, with the bottom-right quadrant designated as the region of interest (ROI) by using PowerPoint a technique we had from our previous research^15^. The image processing follows three steps: (1) There were two distinguishable colors, green and brown (rockwool block surface) or white (rockwool bag surface). (2) The two zones segmented easily by the ‘Remove Background’ tool in Microsoft PowerPoint. (3) The process has been tried several times to have the best segmentation. Afterwards, on the rockwool block surface, the diagonal opposite side of the irrigation facility (dripper) was selected as the ROI because it is the best place to understand the influence of irrigation. More details on ROI can be found in the data sources and acquisition protocols section. We took photos after cutting the crop to get a better ROI and finished taking the photos in one go to minimize the environmental impact (PPF 313.4 µmol m⁻² s⁻¹), as measured using a spectroradiometer (LI-180; LI-COR Biosciences, Lincoln, NE, USA). This analysis enabled the categorization of sensors into two distinct categories: those exhibiting high algae colonization (≥ 90% coverage) and those demonstrating minimal coverage (≤ 10%). Tomato yield data were collected on four scheduled harvest dates, with measurements taken for fruit count and weight of each plant to assess the relationships between sensor reliability, environmental parameters, and crop productivity. This visual summary provides a clear overview of the study framework, linking all data streams (environmental, algal coverage, and yield) that underpinned the subsequent analysis.

### Study setup

**Fig. 1:**
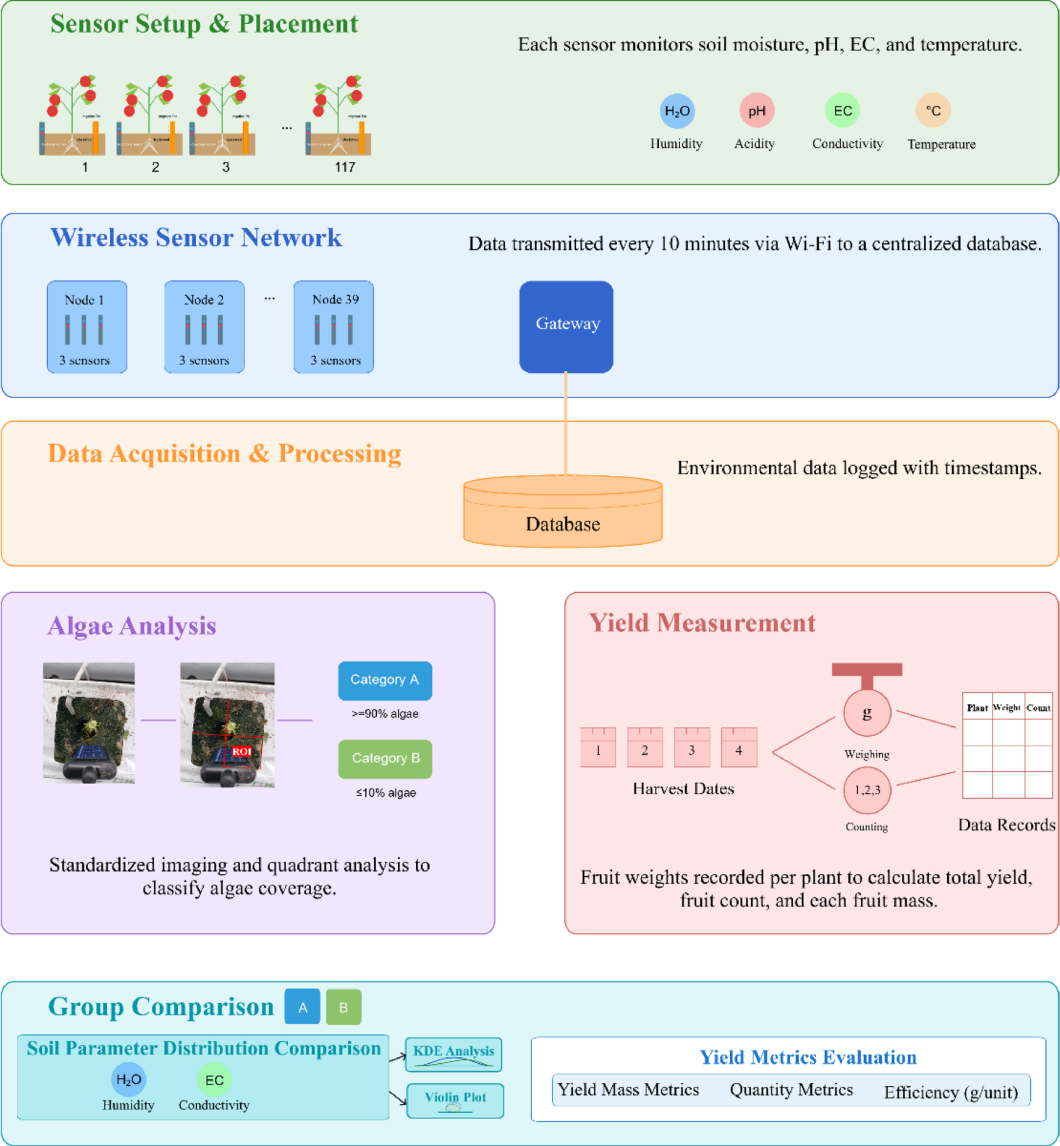
Schematic representation of the study framework for sensor reliability assessment in hydroponic tomato cultivation.

#### Study rationale and hypothesis development

This investigation was initiated following observation of substantial variation in sensor-recorded environmental parameters (humidity, EC, temperature, pH) during the cultivation period, despite uniform greenhouse conditions and identical sensor specifications across all 117 monitoring units. Since sensor functionality testing revealed no equipment malfunctions, alternative explanations for the data variation were sought. Post-cultivation visual inspection of rockwool block surfaces revealed marked differences in algal coverage patterns across sensors. Given that algal proliferation is influenced by the availability of minerals and moisture^16^, we hypothesized that algae coverage ratios might serve as indicators of differential nutrient solution distribution within the substrate—with high algae coverage indicating areas where nutrient solution effectively reached, and low coverage suggesting insufficient solution penetration. This hypothesis predicts that sensors with different algae coverage patterns would record systematically different environmental parameters, particularly humidity and EC, reflecting localized differences in substrate moisture and nutrient availability rather than sensor errors. To test this hypothesis, sensors were retrospectively categorized based on quantified algae coverage (≥ 90% vs. ≤ 10%), and environmental parameters were compared between categories to determine whether algae coverage patterns could explain the observed sensor data variation.

**Fig. 2:**
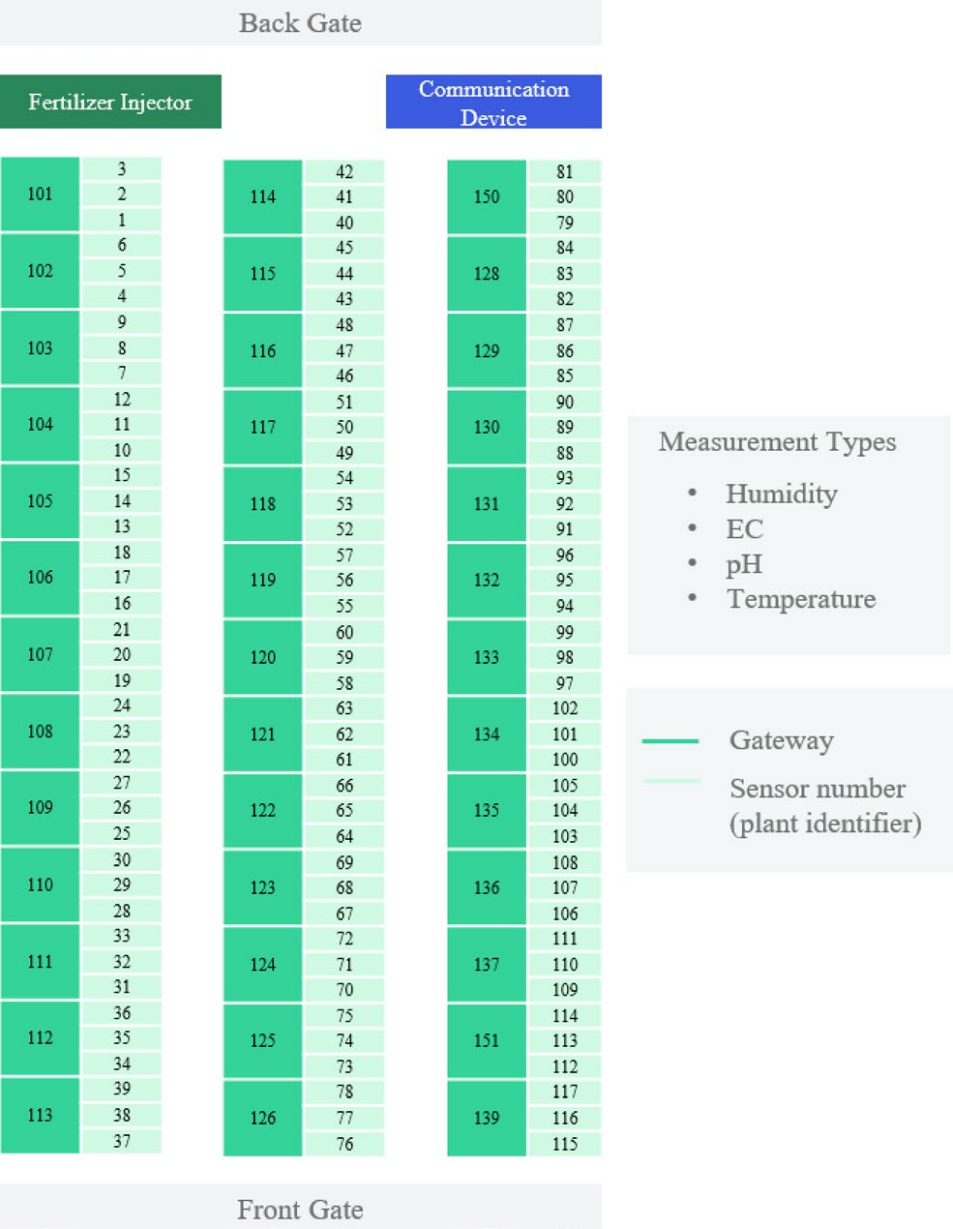
Experimental setup farm layout.

The study setup comprised 117 tomato plants systematically arranged between the front and back gates, with sensor identifiers sequentially allocated from 1 to 117, as illustrated in Fig. 2. Each plant was subjected to an extensive sensor array to measure root-zone moisture, EC, pH, and temperature in the root zone. The system architecture integrated three sensors to employ a single gateway, thereby establishing a distributed network of communication nodes throughout the cultivation area (36°40′00.1″ N, 126°51′46.8″ E). Data gathering occurred over 3 months, from July 18 to October 16, 2024, utilizing automated sensor recording devices for continuous environmental monitoring.

The research team evaluated tomato yield on four specific dates: September 23, September 28, October 7, and October 16, facilitating comprehensive monitoring of crop productivity. After cultivation, algal development on each sensor surface was documented to analyze distribution patterns and their correlations with sensor dependability.

#### Data sources and acquisition protocols

The data collection system integrated three main data streams via a hierarchical network architecture, wherein biosensors and root zone sensors were linked to wireless nodes that relayed data through a gateway utilizing Ethernet protocols to a centralized database. This infrastructure generated environmental data, including pH levels, EC values, and root zone temperatures, at 10-min intervals. Each measurement was complemented by a distinct date timestamp for accurate spatiotemporal tracking. The study also involved photographic records of algal proliferation on individual plant sensor units, illustrating the distribution patterns of algal development on each sensor surface. The methodology included collecting crop yield data at four designated harvest periods (September 23 and 28, October 7 and 16) to facilitate a temporal analysis of productivity measures during the cultivation period.

**Fig. 3:**
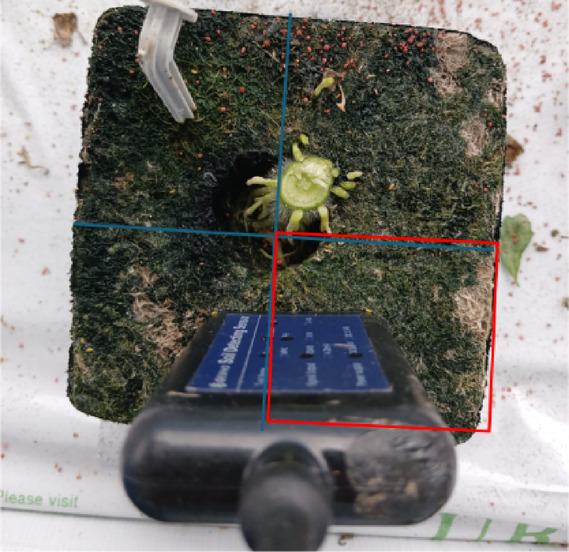
Sensor 10 sample photo.

The photographic documentation procedure established a standardized approach for analyzing the algal distribution on the sensor surfaces, as shown in Fig. 3. Each sensor was systematically photographed, with the camera positioned perpendicularly to the sensor surface to ensure consistent image quality. Following image acquisition, each photograph was divided into four equal quadrants using a standardized grid overlay, with analysis concentrated on the bottom-right quadrant as the ROI. This study assessed the algal coverage ratios within this designated ROI, enabling the categorization of sensors into two distinct groups based on coverage percentage: those exhibiting greater than 90% coverage and those showing approximately 10% coverage. Each image included the gateway identification number of the sensor for precise tracking and documentation, thereby enabling the systematic analysis of algal distribution patterns in relation to the sensor performance metrics.

A systematic documentation method was implemented as a yield evaluation technique to verify tomato production throughout the cultivation period. This method enabled the assignment of a unique identity to each plant through sensor numbers, thereby establishing traceable records for subsequent harvests.

Each fruit from the harvest was weighed individually, and the weights were recorded in a standardized spreadsheet. Harvest dates were scheduled based on four specific time points: (1) September 19 (S1); (2) September 23 (S2); (3) September 28 (S3); and (4) October 17 (S4). Yields from each harvest date were indexed into columns to maintain temporal precision. The methodology incorporated automation to streamline the efficiency of calculating total harvest weight (g), total quantity of harvested fruit (amount), and average fruit mass per plant (g per plant).

#### Analytical approach

This study employed a binary classification technique to categorize sensors based on quantifiable algal coverage patterns and established two distinct groups for comparative analysis. The first group consisted of sensors that exhibited extensive algal proliferation, with coverage exceeding 90% of the sensor surface area (Category A). In comparison, the second group demonstrated minimal algal formation, covering approximately 10% of the sensor surface area (Category B). This categorical technique enabled the systematic investigation of potential correlations between algal coverage patterns and critical environmental parameters, namely root-zone humidity and EC, while allowing simultaneous analysis of the relationship between algal distribution and tomato yield metrics across both sensor groups. All statistical analyses were performed using Python 3.10 with the following libraries: pandas for data manipulation, scipy.stats for statistical tests, numpy for numerical computations, matplotlib and seaborn for data visualization. Correlation analyses were conducted using Pearson and Spearman correlation coefficients. Mann-Whitney U tests were implemented to assess distributional differences between categories. Kernel density estimation (KDE) and violin plots were generated using seaborn for data visualization.

## Results

### Algorithm coverage distribution

**Fig. 4:**
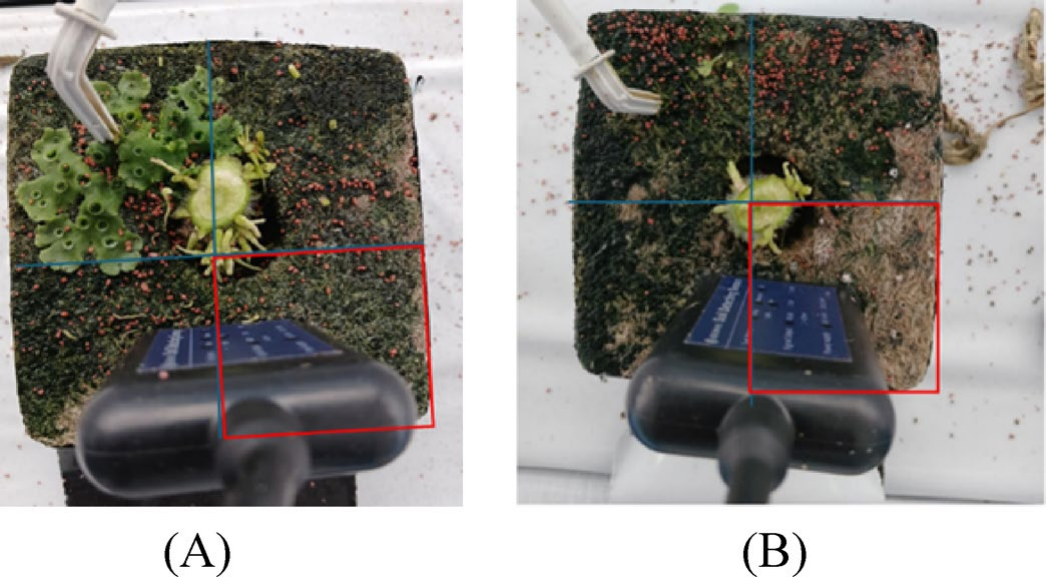
Algal coverage patterns between experimental categories (A) representative high coverage specimen (≥ 90%, Category A) and (B) low coverage specimen (≤ 10%, Category B), illustrating distinct colonization patterns across sensor surfaces.

This study examined the extent of algal coverage across different regions using 117 sensors. The coverage percentages observed ranged from 0% to 100%. As illustrated in Fig. 4, a concise classification system based on algal growth characteristics was used. Group A (*n* = 22) comprised sensors exhibiting high algal coverage of 90% or more. This group included Sensors 9, 13, 23, 27, 29, 49, 53, 56, 57, 62, 85, 86, 87, 91, 92, 93, 94, 97, 102, 103, 107, and 109, exhibiting coverage percentages ranging from 90% to 100%. Conversely, Group B (*n* = 17) comprised sensors characterized by minimal presence (approximately 10%), including Sensors 2, 6, 12, 15, 18, 24, 26, 34, 38, 67, 68, 72, 75, 76, 78, 84, and 90, exhibiting coverage percentages between 0% and 10%.

### Environmental data distribution

**Fig. 5:**
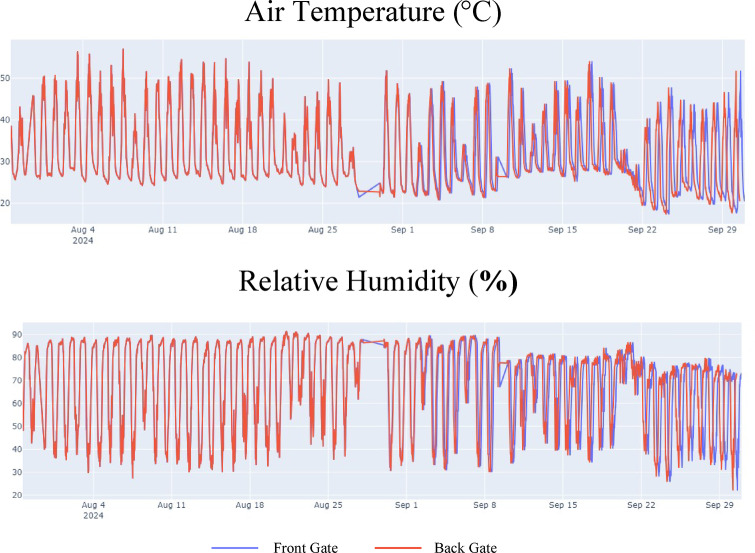
Controlled greenhouse air temperature and relative humidity.

**Fig. 6:**
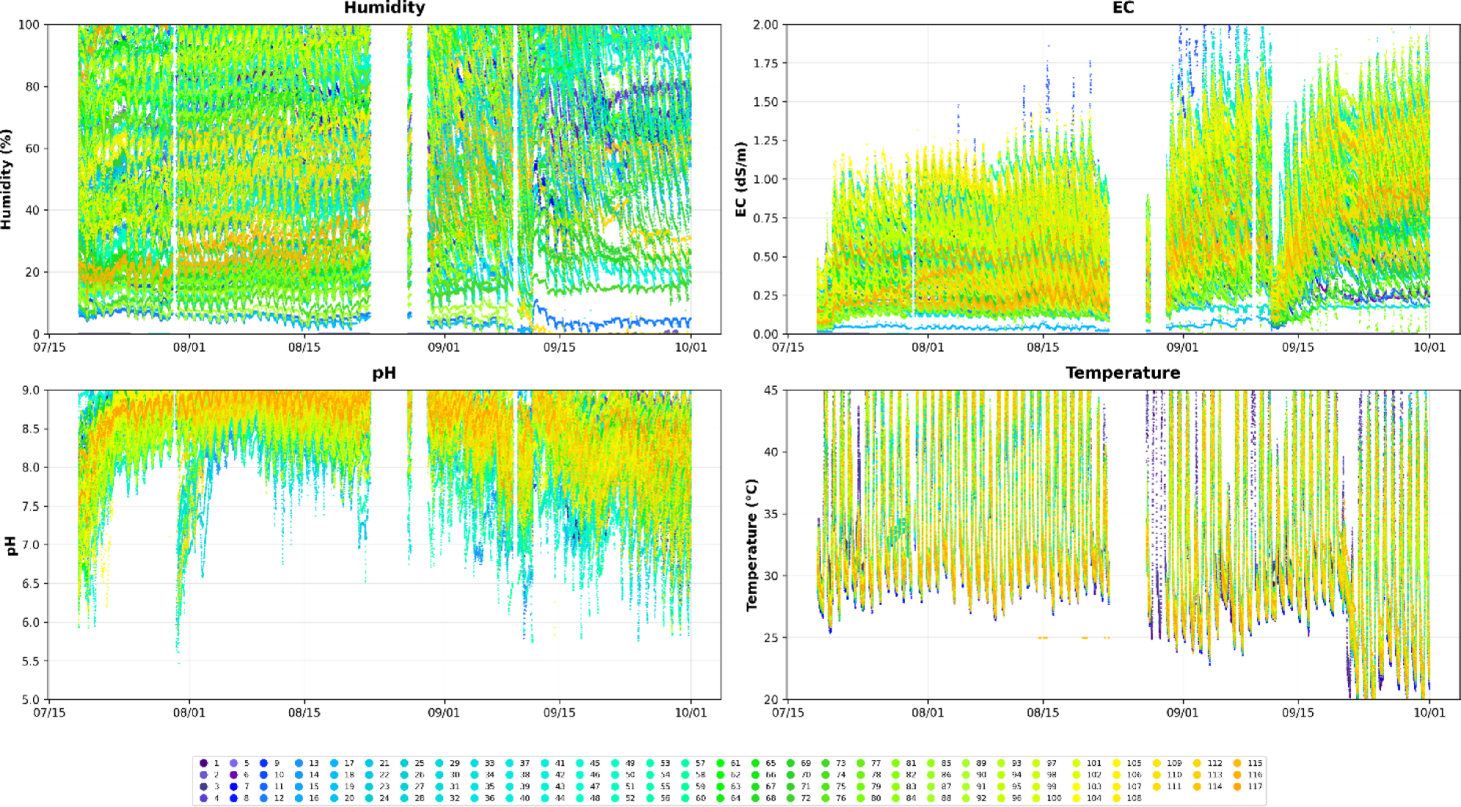
Comprehensive visualization of root zone condition monitoring data.

Figure 5 illustrates the distributions of the controlled greenhouse air temperature and relative humidity. The data indicated that the greenhouse environment was characterized by high temperatures and humidity levels. Occasionally, tropical nights were also observed. However, a sensor malfunction occurred between August 25 and September 1, 2023, resulting in constant temperature and relative humidity readings during this period. Figure 6 shows the distribution of the root zone characteristics. The trend in the root-zone temperature mirrored that of the air temperature; however, the root-zone humidity showed a very large variation.

### Root zone parameter analysis

#### Correlation statistics by category

**Fig. 7:**
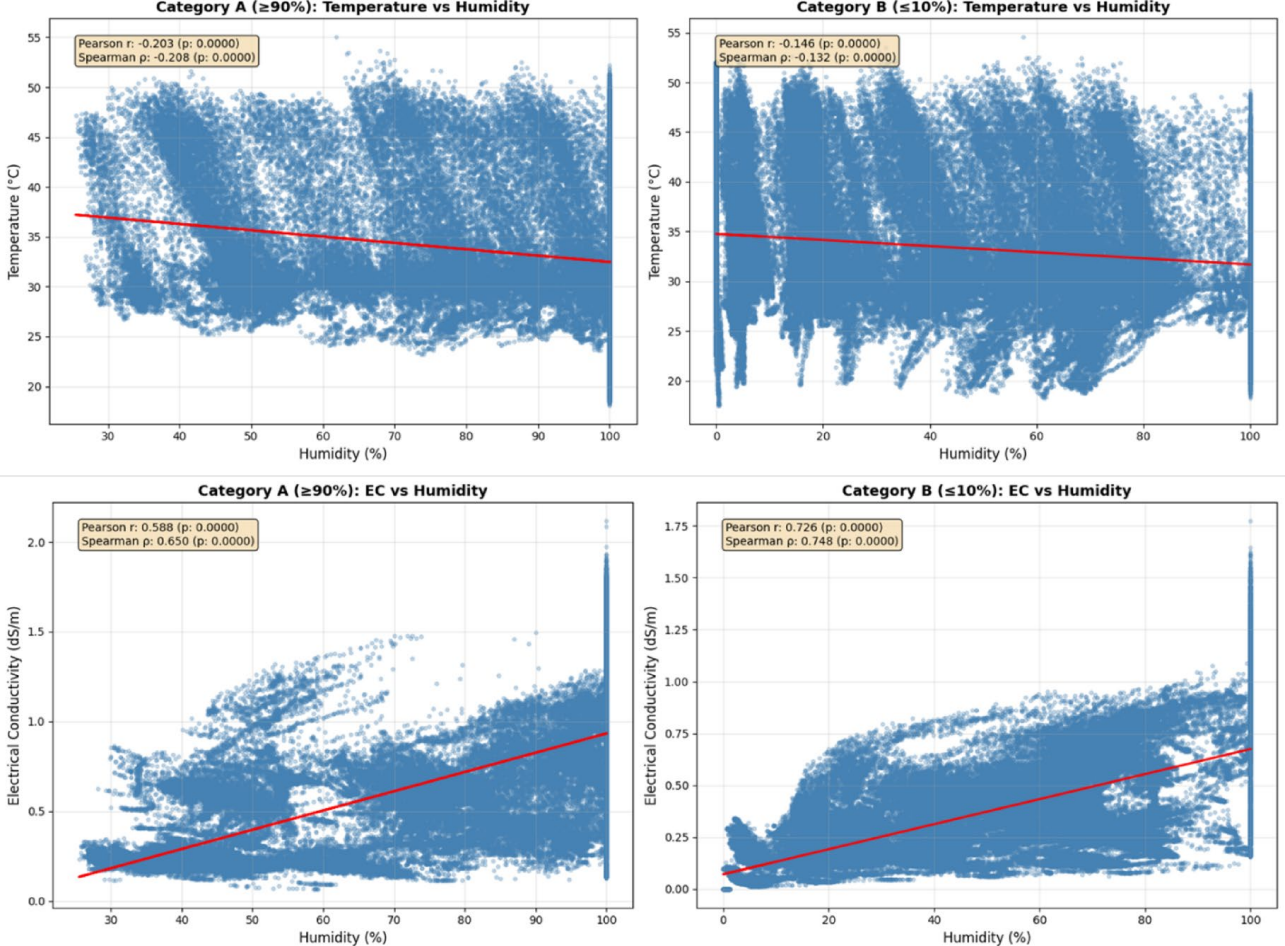
Correlation analysis between root zone parameters across high and low moisture environments.

Figure 7 presents scatter plots accompanied by regression lines (red) that compare the relationships between root-zone environments in the two distinct categories. Figure 7a, b shows a weak negative correlation between root zone temperature and humidity in both Category A (*r* = −0.203) and Category B (*r* = −0.146) plants. Figure 7c, d indicates a strong positive correlation between root-zone EC and humidity for both Category A (*r* = 0.588, ρ = 0.650) and Category B (*r* = 0.726, ρ = 0.748). All correlations were statistically significant (*p* < 0.001). These relationships highlight the effects of moisture content on root-zone temperature regulation and nutrient availability in agricultural growth media under different hydration conditions.

#### Root zone humidity distribution analysis

**Fig. 8:**
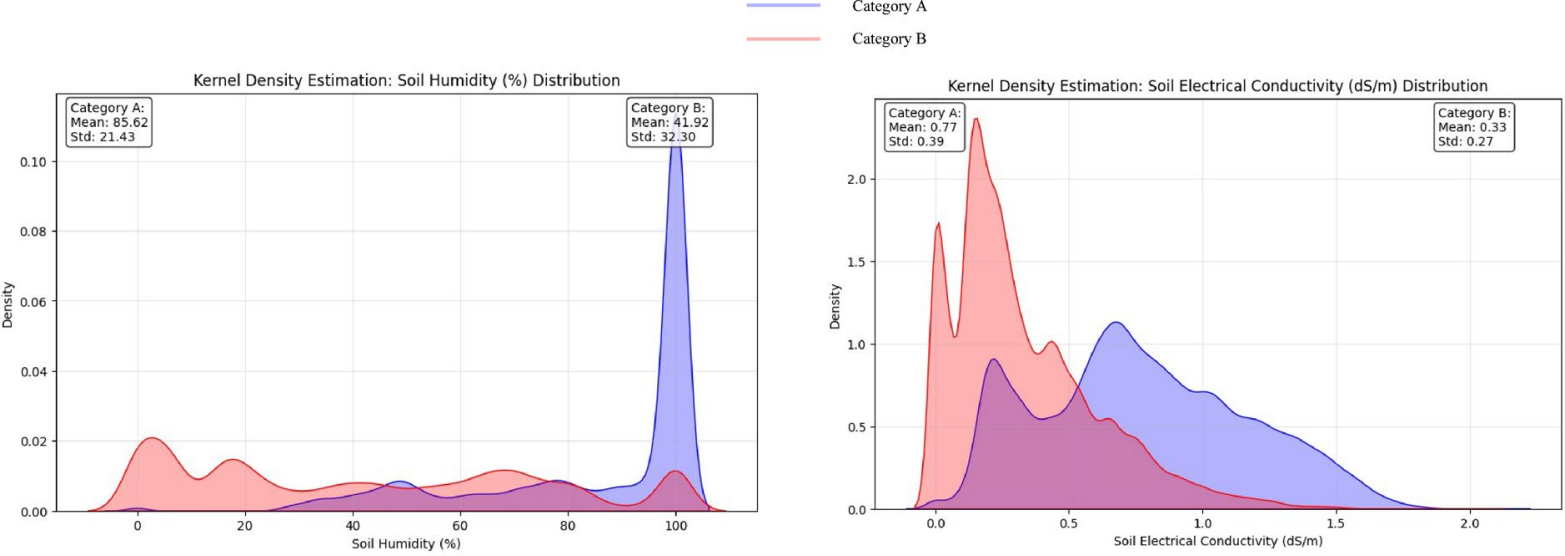
Root zone humidity and electrical conductivity (EC) kernel estimation density (KDE) graph.

**Fig. 9:**
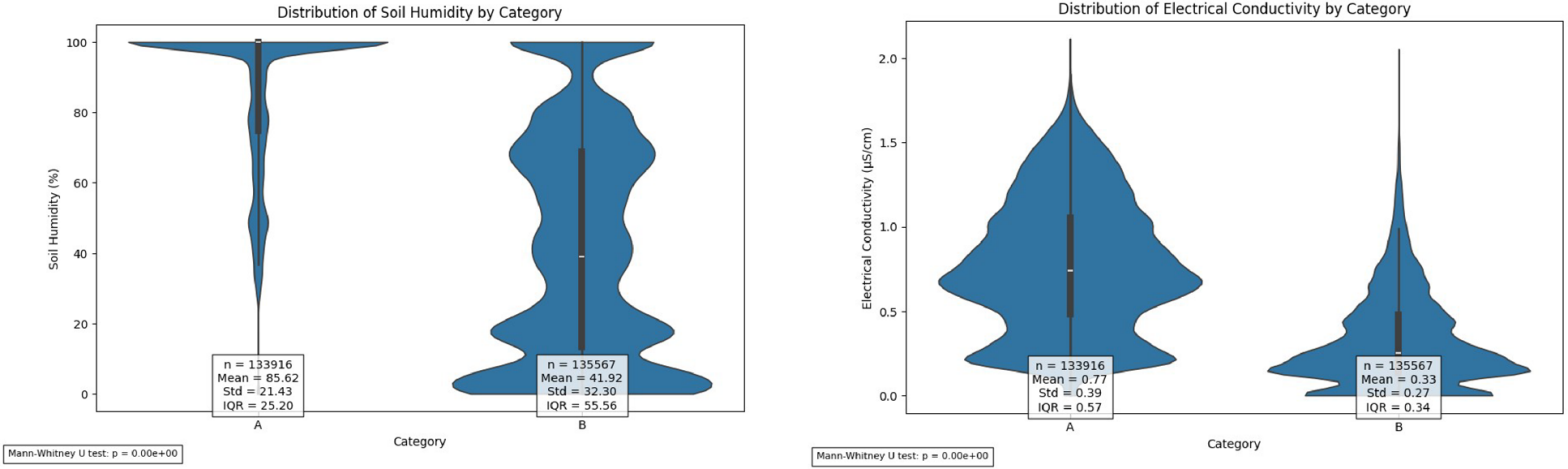
Violin plot for the comparative distribution between the two groups.

The comparative analysis of root-zone parameters between the two categorical groups revealed distinct distributional characteristics, as demonstrated by kernel density estimation (KDE) and violin plot visualization (Figs. 8 and 9). This study employed dual analytical methodologies to analyze the distribution patterns of root-zone humidity and EC measurements across the groups. The KDE analysis revealed considerable bimodality in the root-zone humidity distribution, with Category A exhibiting a sharp density peak near 100% humidity (mean = 85.62%, standard deviation (SD) = 21.43).

This distribution pattern is further corroborated by the violin plot morphology, which reveals a distinctive narrow waist with concentrated density at relatively high humidity levels [interquartile range (IQR) = 25.20].

Conversely, Category B exhibited a more diffuse distribution (mean = 41.92%, SD = 32.30), characterized by a substantially greater spread (IQR = 55.56) and multiple modal regions across the measurement spectrum, as evidenced by both analytical visualizations.

#### Electrical conductivity analysis

The EC measurements revealed distinct distributional characteristics between the categories. The KDE visualization revealed a right-skewed distribution for Category A (mean = 0.77 dS/m, SD = 0.39), with increased density observed in higher conductivity regions (0.5–1.5 dS/m). The corresponding violin plot quantified this variation with an expanded interquartile range (IQR = 568.00), indicating a substantial measurement distribution. Category B demonstrated a more compressed distribution (mean = 0.33 dS/m, SD = 0.27, IQR = 0.34), with the KDE plot revealing multiple low-amplitude modes mainly concentrated in the 0–0.5 dS/m range. The corresponding violin plot confirmed this constrained measurement distribution through its narrowed morphology relative to that of Category A. The statistical significance of these distributional differences was confirmed through Mann‒Whitney U tests (*p* < 0.05) for both parameters, providing robust evidence for systematic variation in environmental conditions between high- and low-algal coverage zones.

These findings collectively indicate systematic variations in both humidity and EC between algal coverage categories and suggest an association between environmental parameters and algal proliferation patterns in agricultural monitoring systems. Moreover, the trend was presented even in the early stages of cultivation without algae, indicating that algae did not directly affect the sensor errors (Fig. s1).

**Table 1:** Descriptive statistics and statistical comparisons of Root-Zone environmental parameters by algal coverage Category.

Parameter	Category A (≥ 90% coverage)	Category B (≤ 10% coverage)	*p*-value
Sample size (n)	22	17	
Environmental parameters			
Humidity (%)			
Mean ± SD	85.62 ± 21.43	41.92 ± 32.30	< 0.001
Median	100.00	37.30	
IQR	25.20	54.54	
Range	25.51–100.00	0.00–100.00	
EC (dS/m)			
Mean ± SD	0.77 ± 0.39	0.33 ± 0.27	< 0.001
Median	0.75	0.24	
IQR	0.57	0.32	
Range	0.06–2.11	0.00–1.77	
Temperature (°C)			
Mean ± SD	33.35 ± 6.44	33.51 ± 6.67	0.013
Median	31.42	*31.38*	
IQR	8.14	*8.64*	
Range	18.10–55.03	17.52–54.56	
pH			
Mean ± SD	8.45 ± 0.50	8.75 ± 0.49	< 0.001
Median	8.54	8.76	
IQR	0.57	0.55	
Range	5.69–9.57	7.04–9.74	
Correlation analysis			
Temperature × Humidity (Pearson r)	−0.203	−0.146	< 0.001
EC × Humidity (Pearson r)	0.588	0.726	< 0.001
EC × Humidity (Spearman ρ)	0.650	0.748	< 0.001

*p* < 0.05; *p* < 0.001; ns = not significant.

Table 1 presents comprehensive descriptive statistics and statistical comparisons for all measured environmental parameters (corresponding to Figs. 6–9). The analysis confirms significant differences between algal coverage categories for humidity (*p* < 0.001), EC (*p* < 0.001), and pH (*p* < 0.001), while temperature showed a statistically significant but small difference (*p* = 0.013).

#### Production metric analysis

The quantitative assessment of agricultural production parameters graded by algal coverage classification revealed nuanced patterns across multiple performance indices. Table 2 and Fig. s2 present a comprehensive analysis encompassing mass-based measurements, quantity indices, and efficiency metrics for both high-coverage (Category A, ≥ 90%) and low-coverage (Category B, ≤ 10%) conditions.

**Table 2:** Comprehensive analysis of production metrics by algae coverage classification.

Performance metric	Category A (≥ 90% coverage)	Category B (≤ 10% coverage)
Sample size (n)	22	17
Production mass metrics		
Mean production (g)	1,680.45 ± 345.66	1,602.06 ± 341.44
Median production (g)	1,662.50	1,615.00
Production range (g)	810–2555	1,010–2.220
Production IQR (g)	310.00	470.00
Production quantity metrics		
Mean quantity (units)	9.95 ± 2.21	9.65 ± 2.57
Median quantity (units)	10.00	10.00
Quantity range (units)	5–15	5–15
Quantity IQR (units)	3.00	2.00
Efficiency metrics		
Production coefficient (g/unit)	168.89	166.02

^1^ Production Coefficient = Mean Production/Mean Quantity.

Mass-based production analysis demonstrated minimal divergence in the central tendency measures between the categories. Category A (*n* = 22) presented a mean production of 1,680.45 ± 345.66 g, with a median of 1,662.50 g, whereas Category B (*n* = 17) presented comparable metrics, with a mean of 1,602.06 ± 341.44 g and a median of 1,615.00 g. However, the distribution characteristics revealed distinct patterns, with Category A exhibiting a broader production range (810–2,555 g) and more concentrated dispersion (IQR = 310.00 g) compared with Category B, which exhibited a narrower range (1,010–2,220 g) and greater interquartile variation (IQR = 470.00 g).

The quantity metrics demonstrated remarkable consistency across the categories. Both conditions maintained identical median values (10.00 units) and range boundaries (5–15 units). The mean quantities showed minimal variation (Category A: 9.95 ± 2.21 units; Category B: 9.65 ± 2.57 units), with Category A demonstrating marginally greater dispersion (IQR = 3.00 units) than that of Category B (IQR = 2.00 units).

The production coefficient analysis revealed minimal variation in system efficiency under various conditions (Category A: 168.89 g/unit; Category B: 166.02 g/unit), implying that algal coverage exerted a limited effect on production efficiency. The marginal differential of 2.87 g/unit (1.72%) indicated robust system performance, regardless of the extent of algal proliferation.

These findings indicate that despite variations in algal coverage, the agricultural system maintained consistent production characteristics across both mass- and quantity-based metrics, indicating robust operational resilience to varying environmental conditions.

## Discussion

In this study, we investigated the effect of algal formation on the reliability of sensor data in hydroponic tomato cultivation using rockwool. The environmental characteristics of the root zone result from the rockwool, which possesses a porous structure and high-water retention capacity. The relationship between the temperature and humidity in rockwool demonstrates the cooling effect of fertigation^17^. In contrast, the EC and humidity were strongly and positively correlated because of minerals in the nutrient solution. Because these results are similar to the general characteristics of commercial soil, we believe that rockwool provides favorable conditions for both crop and algal growth^18^.

The bottom-right quadrant, being the most distant from the nutrient solution supply point, was designated as the ROI. Based on the algal coverage ratio, two categories were established: Category A (≥ 90% coverage) and Category B (≤ 10% coverage). As algae lack vascular tissues, they are unable to regulate water and solutes independently, making them highly dependent on environmental factors^19^. Algae absorb moisture and nutrients across their entire surface and thrive only in areas where water is directly available in a drip fertigation system^12^. Previous studies have shown that algal biomass increases when abundant external moisture is available for absorption^20^. Therefore, a high algal coverage ratio signifies that the nutrient mixture from the dripper effectively permeated the edges of the rockwool.

In this study, the sensor-measured humidity and EC values were higher for Category A than those for Category B (Figs. 4 and 5). This aligns with the environmental characteristics conducive to active algal proliferation^21^. Conversely, in Category B, the nutrient mixture failed to adequately reach the sensor location, resulting in lower humidity and EC values, with the algal coverage remaining below 10%. The observed differences in the environmental parameters between Categories A and B suggest that the nutrient mixture was unevenly distributed across the rockwool substrate in the drip fertigation system. These findings indicate that algal coverage can serve as an indirect indicator of localized environmental characteristics, specifically humidity and EC levels, and may also be useful for assessing the reliability of sensor data.

Despite variations in the root zone parameters, the production metric analysis revealed minimal differences between the two categories (Table 1). Previous study also reported that tomato plants could maintain their yield even under certain levels of environmental stress^22^. These findings suggest that tomatoes exhibit high levels of resilience to environmental fluctuations and sustain consistent production levels despite algae-induced changes. However, sensor data may not accurately reflect actual environmental differences and can be subject to measurement errors due to environmental conditions.

Previous studies have demonstrated that the humidity and EC levels in hydroponic systems can affect tomato mass and yield^23,24^. In this study, significant correlations were observed between algal coverage and sensor data reliability, with differences in humidity and EC values recorded between Categories A and B. However, despite these environmental differences, no significant variation in production metrics was observed between the two categories. This can be attributed to the extension of tomato roots throughout the rockwool substrate from the core to the periphery, which ensures consistent nutrient uptake^25^. Additionally, the sensors collected data from locations more distant from the drippers than those from the plants, potentially leading to an inaccurate representation of the environmental factors influencing actual crop growth. This means that the nutrient solution reached the plants but not the sensor located further away. Or, the plant roots may have obstructed the nutrient solution’s movement.

Consequently, sensor measurements may not directly reflect changes in crop productivity and can be susceptible to measurement errors influenced by environmental factors. While previous studies have focused primarily on sensor malfunctions or data transmission issues^26–28^, future research should explore instances in which sensors operate normally yet yield inaccurate readings owing to extraneous environmental factors. Achieving this goal requires identifying data differences based on sensor design and subsequently analyzing the relationship between crop production and sensor data under various environmental conditions. Based on these studies, improved sensor designs, and maintenance strategies may be developed that enable the collection of more reliable data and improve the accuracy of crop yield monitoring.

## Conclusion

Sensor reliability is a critical factor in agricultural data collection. This study emphasizes the importance of sensor reliability in hydroponic systems and evaluates sensor data by examining the environmental parameters that influence algal growth. A correlation was observed between algal coverage and the environmental parameters (humidity and EC) as measured by the sensors. This study demonstrated algae coverage should be interpreted as an indirect indicator of localized environmental conditions rather than as a causal factor or sensor malfunction. However, the findings indicate that data errors influenced by environmental factors may occur during sensor measurements, highlighting the need for improved sensor designs and maintenance strategies to collect reliable agricultural data. Data collection that considers sensor reliability is anticipated to enable more accurate agricultural monitoring.

## Supplementary Information

Below is the link to the electronic supplementary material.

Supplementary Material 1

## Data Availability

The datasets generated and analyzed during the current study are publicly available in this repository https://github.com/saksonita/smartfarm\_Dataset.
