# Supplementary material for: Reliability assessment of agricultural sensors evaluated through algal coverage in hydroponic tomato production systems
Source: Sci Rep. 2026 Feb 12;16:8529. doi: 10.1038/s41598-026-38555-y (PMC12976035; doi:10.1038/s41598-026-38555-y)
Supplement: Supplementary file 1 — Supplementary Material 1 [file 41598_2026_38555_MOESM1_ESM.docx]

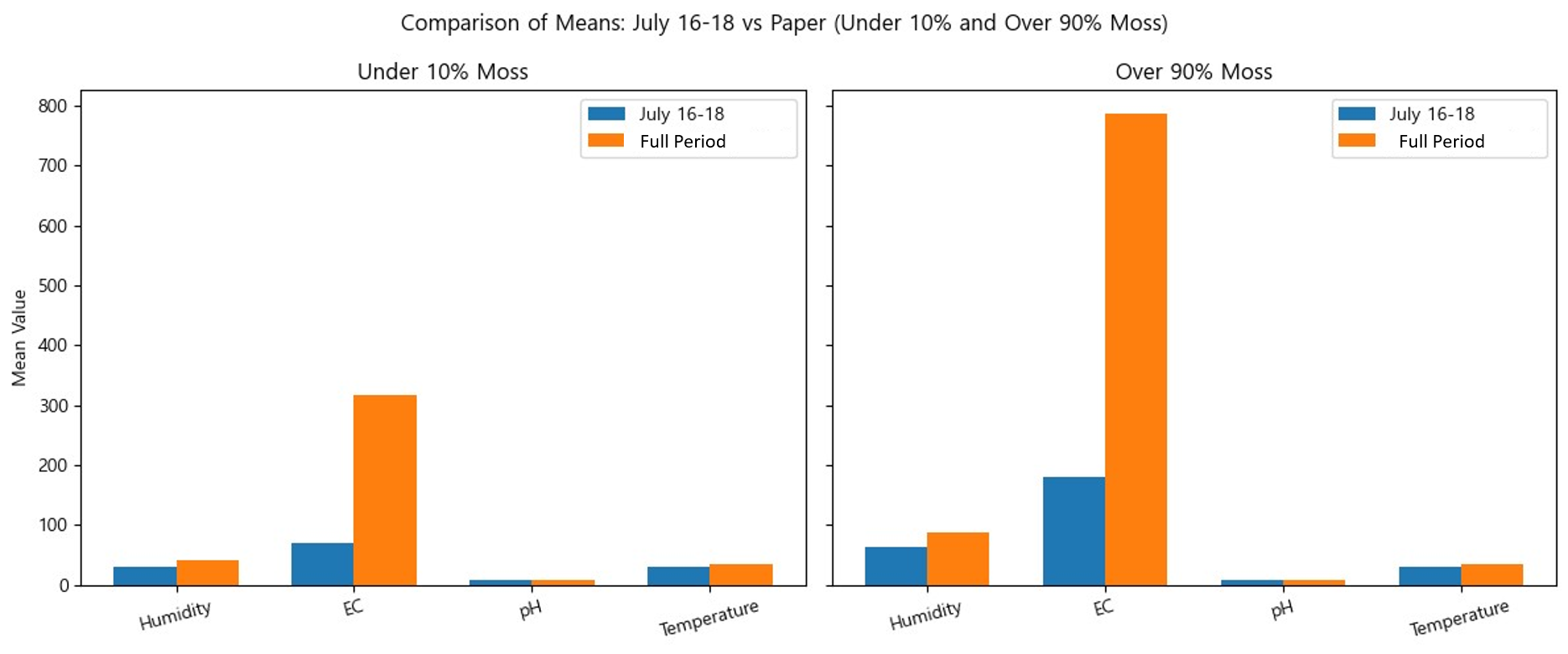


**Supplementary Fig. 1** The comparison sensor readings before and after applying the nutrient solution.

**Supplementary Fig. 2** The information of tomato fruit production.
